# Neighborhood variation in unsolved homicides: a retrospective cohort study in Indianapolis, Indiana, 2007–2017

**DOI:** 10.1186/s40621-020-00287-6

**Published:** 2020-12-01

**Authors:** Lauren A. Magee, J. Dennis Fortenberry, Wanzhu Tu, Sarah E. Wiehe

**Affiliations:** 1 O’Neill School of Public and Environmental Affairs, Indiana University Purdue University Indianapolis, 801 W. Michigan Street, Indianapolis, Indiana 46202 USA; 2 Department of Adolescent Medicine, Indiana University School of Medicine, 410 W. 10th Street, Suite 1000, Indianapolis, Indiana USA; 3 Department of Biostatistics, Indiana University School of Medicine, 410 W. 10th Street, Suite 3000, Indianapolis, Indiana USA; 4 Department of Pediatrics, Indiana University School of Medicine, 410 W. 10th Street, Suite 2000, Indianapolis, USA

**Keywords:** Homicides, Urban violence, Epidemiology

## Abstract

**Background:**

Homicide is a widely acknowledged public health problem in the United States. The majority of homicides are committed with a firearm and have long-term health consequences for family members and entire communities. When left unsolved, violence may be perpetuated due to the retaliatory nature of homicides. Improving homicide clearance rates may help prevent future violence, however, we know little about the community-level social dynamics associated with unsolved homicides.

**Methods:**

This study examines the individual-and-community-level social processes associated with low homicide clearance rates in Indianapolis, Indiana between 2007 and 2017. Homicide clearance is the primary outcome, defined as if a perpetrator was arrested for that homicide case between 2007 and 2017. Individual-level variables include the victim’s race/ethnicity, sex, and age. Community-level (i.e., census tracts) variables include the number of resident complaints against the police, resident complains of community disorder, income inequality, number of police interactions, and proportion of African American residents.

**Results:**

In Indianapolis over a 11-year period, the homicide clearance rate decreased to a low of 38% in 2017, compared to a national clearance rate of 60%. Homicide case clearance was less likely for minority (OR 0.566; 95% CI, 0.407–0.787; *p* < 0.01) and male (OR 0.576; 95% CI, 0.411–0.807; *p* < 0.01) victims. Resident complaints of community disorder were associated with a decreased odds of case clearance (OR 0.687; 95% CI, 0.485–0.973; *p* < .01)., African American victim’s cases were less likely to be cleared in 2014–2017 (OR 0.640; 95% CI, 0.437–0.938; *p* < 0.05), compared to 2007.

**Conclusions:**

Our study identified differences in neighborhood social processes associated with homicide clearance, indicating existing measures on these community factors are complex. Programs aimed at improving signs of community disorder and building community engagement may improve neighborhood clearance rates, lower violence, and improve the health of these communities.

## Introduction

Homicide is a widely acknowledged public health problem in the United States (Adhia et al., 2019; Culyba et al., 2016). The homicide rate in the United States is higher than other high-income nations and the firearm homicide rate is nearly 25 times higher compared to other high-income nations (Grinshteyn & Hemenway, 2016). In the United States, homicide rates and suicide rates are higher in states with higher rates of firearm ownership (Miller et al., 2007a; Siegel et al., 2013; Miller et al., 2007b), urban homicides are most often committed with a firearm (Puzzanchera, 2019), and firearms contribute to a number of unintentional deaths each year (Hemenway & Solnick, 2015). Homicide rates have increased dramatically in recent years and many are left unsolved contributing to the continued health inequality of residents within urban communities. This study explores the individual-and-community characteristics associated with neighborhood-to-neighborhood variation in unsolved homicides.

The largest one-year increase in homicides in the United States since 1968 occurred between 2014 to 2015, with an 11.4% increase, and rates jumped another 8.2% from 2015 to 2016 (Rosenfeld et al., 2017). This homicide increase followed the events of two high-profile officer-involved killings of Michael Brown in Ferguson, Missouri and Eric Garner in Staten Island, New York in 2014. Given the timing of these events and the increase in overall crime and specifically homicides, many tied the rise to the de-policing of certain proactive activities, which have been deemed the “Ferguson effect” (Rosenfeld et al., 2017; Rosenfeld & Wallman, 2019). Research into the association between the Ferguson effect indicates some departments did engage in de-policing activities, such as fewer traffic stops, searches, and arrests within African American communities, however, these changes in police activity were not associated with increases in overall crime rates or the increase in homicides (Rosenfeld & Wallman, 2019; Shjarback et al., 2017; Pyrooz et al., 2016). Despite a lack of association with increased crime rates, research examining community member attitudes towards the police does support a comprised police legitimacy payable to the Ferguson effect (Gaston et al., 2019). These findings highlight the importance of the relationship between the community and the police; as residents who view the police as illegitimate are less likely to cooperate with police (Tyler, 2004). Homicide cases are more likely to be solved when witnesses and residents provide information to the police (Litwin, 2004; Riedel, 2008), however, noncooperation can be driven by fear (Riedel, 2008), or lack of trust in the police (Regoeczi & Jarvis, 2013). Unsolved homicides may continue the cycle of violence through retaliation (Cook & Ludwig, 2019), contributes to the trauma that family members of homicide survivors’ experience (Wellman & Meitl, 2020; Stretesky et al., 2016; Stretesky et al., 2010; Simmons et al., 2014), and arguably to the larger communities, as homicides spread across communities much like an infectious disease (Zeoli et al., 2012).

Low homicide clearance rates (i.e., homicide arrest rates within communities) are associated with economic disadvantage, residential instability, and lack of collective efficacy (Regoeczi & Jarvis, 2013; Mancik et al., 2018; Litwin & Xu, 2007). There are mixed findings between homicide clearance and either victim race or neighborhood racial composition (Litwin & Xu, 2007; Puckett & Lundman, 2003; Lundman & Myers, 2012; Petersen, 2017). Furthermore, neighborhood social processes, such as views of community disorder (Skogan, 1992) and police legitimacy (Kirk & Matsuda, 2011) differ across communities and may influence homicide clearance. Broken windows theory posits that community disorder indicates signs of physical decay and social unrest within a community, which may indicate residents are less willing to act as guardians of the neighborhood and lead to higher crime rates (Wilson & Kelling, 1982). Communities with higher levels of disorder may be more likely to be policed through broken windows policing, which has shown some success in reducing crime (Braga et al., 2015). This form of policing, however, has also shown to cause harm to many communities of color (Sherman & Eck, 2002; Skogan, 2006a), as it increases interactions between police and residents for lower level offenses, has led to more complaints against the police (Sherman & Eck, 2002; Kamalu & Onyeozili, 2018), and can lead to views of less police legitimacy (Kamalu & Onyeozili, 2018). Police legitimacy can be gained or lost based on resident interactions with the police (Tyler & Fagan, 2008). If the resident feels treated fairly then s/he is more apt to cooperate with the police and view police work as legitimate in future interactions; the contrary is also possible (Tyler & Fagan, 2008). Therefore, understanding the association between resident complaints of disorder, complaints against the police, and homicide clearance may be an important first step in preventing future violence within the community and contributing to improved health equity across urban communities (Cook & Ludwig, 2019).

Prior studies have extensively studied the homicide event circumstances (i.e., motive, weapon, detective, etc.), however, very few studies have explored the community social processes associated with homicide clearance. In the summer of 2018, the *Washington Post* conducted an in-depth analysis of over 52,000 homicides across the United States (Lowery et al., 2018). The authors found areas within major cities where homicides were common but arrests for those homicides were rare and deemed these areas “pockets of impunity” (Lowery et al., 2018). The police attribute the lack of arrest in these areas to poor relationships with residents and witnesses being fearful of potential retaliation. Residents within those communities and families of the victims blame the police and their apathy in solving these homicides. Both agree that unsolved homicides continue a cycle of violence within these neighborhoods (Lowery et al., 2018), however, police and residents may have different ideas of why unsolved homicides leads to more violence. One of the cities highlighted in the article was, Indianapolis, Indiana; which has experienced an increase in homicides over the past 3 years. Therefore, the purpose of this study is to examine the individual-and-community level social processes associated with low homicide clearance within Indianapolis neighborhoods. Social processes associated with noncooperation during a homicide investigation could be a key determinant in understanding the cycle of violence, the recent increase in homicides, and reflect the overall health of a community.

## Methods

This is a retrospective cohort study of all homicide events that occurred within Marion County (Indianapolis), Indiana between 2007 and 2017. Data were obtained from public websites provided by the *Washington Post*, the City of Indianapolis, the U.S. Census Bureau, and the Indianapolis Metropolitan Police Department (IMPD). We measured homicide clearance using data from the *Washington Post*. The *Washington Post* collected data on over 52,000 criminal homicides from 50 of the largest cities in the United States. Homicides were identified based on the FBI’s Uniform Crime Reporting (UCR) definitions, and supplemented with death certificate, court records, and medical examiner reports. The *Washington Post* provides the date, location (latitude, longitude, city, state) of the event, clearance status, the victim’s race/ethnicity, sex, and age. These data are more specific at the county level than the national data provided to the FBI through the UCR program. Use of publicly available data has become more common in homicide research, due to the lack of available and accurate data at a national level (Kivisto et al., 2017; Hemenway et al., 2019). Resident complaints against the police and abandoned homes data were obtained from the City of Indianapolis data portal (http://data.indy.gov/). Police 911 calls for service and arrest data were accessed from IMPD. Other community-level measures were available from the U.S. Census Bureau.

### Measures

Outcome measure: The outcome measure is homicide case clearance. Outcome codes were based on three disposition categories: open/no arrest, closed by arrest, and closed without arrest (Riedel, 2008). Closed without an arrest is usually considered exceptionally cleared, and indicates that police have a suspect but do not have enough evidence to make an arrest. Since we were interested in examining homicides where a suspect is unknown, two categories were formed: (1) not cleared and (2) cleared (Regoeczi & Jarvis, 2013).

Race: Victim race/ethnicity (White, African American, Hispanic, other) was combined prior to the Washington Post by the police department or supplemented with death certificate, court records, and medical examiner reports. Race/ethnicity was included at the individual level. Neighborhood racial and ethnic composition was obtained from the 2010 U.S. Census and measured the percent of residents in each census tract reporting race and/or ethnicity as minority (Mancik et al., 2018; Petersen, 2017). The neighborhood racial composition was broken into quartiles, and the three lower quartiles were categorized as having a lower proportion of minority residents, and the upper quartile was categorized as having a higher proportion (Steelesmith et al., 2019).

Community-level measures: Resident complaints of police are formal assertions of procedural injustice and may reflect perceived police illegitimacy (Terrill & Paoline III, 2015). Resident complaints against the police were defined as a complaint made to the Residents’ Police Complaint Office of improper treatment by the officer or that an officer violated department policies during the encounter. Consistent with prior resident complaint studies (Terrill & Paoline III, 2015), we included the number of complaints where residents alleged officers were rude, disrespectful, engaged in substandard performance, neglect of duty, violated rules/laws, used force improperly, or were racial profiling. All categories of resident complaints were combined into an aggregate measure of resident complaints at the census tract level. We then calculated the rate per 100,000 using population-based denominators.

Community disorder combined both social disorder and physical disorder. Social disorder was measured using 911 computer-aided dispatch calls for service data obtained from IMPD. Following prior research, 911 calls for narcotics, public intoxication, disturbances, and loud noise complaints were used as indicators for this measure (Boggess & Maskaly, 2014; O’Brien & Sampson, 2015). Physical disorder was assessed by two data sources: police 911 computer-aided dispatch data and the Indianapolis abandoned home dataset. Resident calls for police response for illegal dumping, vandalism, and abandoned vehicles are indicators of neglect and decay (Skogan, 1992). These 911 call data for social and physical disorder were combined to create a count per census tract. We then calculated the rate per 100,000 using population-based denominators.

Police response to community crimes. Three indicators of police responsiveness were included: police response to firearms use within the community, violent crime rate (minus homicide), and the overall neighborhood arrest rate. Police response to firearms use was measured as the rate of “shots fired” police runs within a neighborhood per 100,000 population, obtained from the IMPD 911 data. Community firearms exposure is a key element of risk of violent victimization (Fagan & Wilkinson, 1998) and homicides committed with a firearm are more difficult to clear (Litwin, 2004; Litwin & Xu, 2007; Jarvis et al., 2017; Rydberg & Pizarro, 2014). The violent crime included Uniform Crime Reports for robbery, aggravated assault, and rape (Investigation FBI, 2004). These crime incidents were combined into one measure and population-based denominator rates per 100,000 were calculated. The neighborhood arrest rate was calculated by the number of neighborhood individuals arrested by the total neighborhood population.

Each of the above community-level measures indicate population rates per 100,000 per census tract. Each measure was categorized into a dichotomous variable, with the three lower quartiles indicating low levels and the upper quartile indicating high levels (Steelesmith et al., 2019).

Income inequality: Income inequality for each neighborhood was assessed by the Gini Index, obtained from the U.S. Census (Elgar et al., 2017). The Gini Index was included as a continuous measure, centered by the grand mean.

Time: We created a variable that included four time periods (2007–2008; 2009–2011; 2012–2014; and, 2015–2017) to control for potential temporal trends.

### Geocoding and geo-aggregation

All homicide event locations, which include victim demographics and year of the event, were obtained from the *Washington Post*. All indicators of community social processes were geocoded using ArcGIS and aggregated to the appropriate census tract (Sonderman et al., 2012) used proxy measure for neighborhood (Petersen, 2017).

### Analysis

We calculated the number of homicides and homicide case clearances per census tract overall and by year. We examined the demographic grouping of victim race/ethnicity, sex, age, and the associated clearance. Following prior work on homicide clearance (Regoeczi & Jarvis, 2013; Petersen, 2017; Braga et al., 2018), we performed a multi-level mixed-effects logistic regression analysis to assess how both victim characteristics and neighborhood context influence the likelihood of homicide case clearance. A census tract-specific random intercept was included in the model to accommodate the potential correlations among homicides within the same census tract. To accommodate the possible spatial correlations, we also performed a sensitivity analysis by including the coordinates into the model in lieu of nesting units within neighborhoods (Wheeler, 2018).

## Results

### Homicides and homicide case clearance rates, 2007–2017

**Fig. 1:**
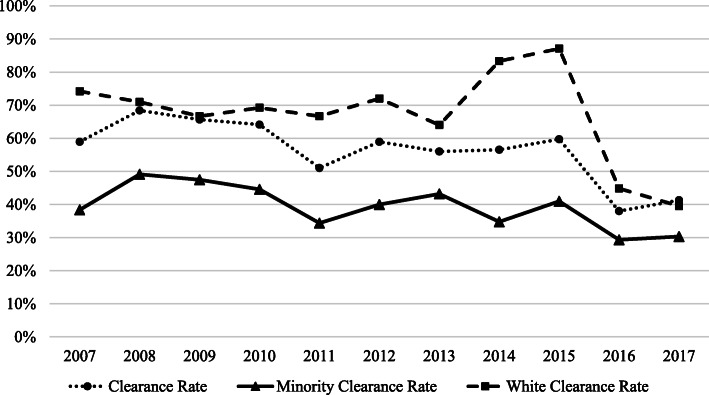
Homicide clearance rate by victim race/ethnicity, 2007–2017

A total of 1320 homicides (mean 7.1 [SD 6.43]) occurred between 2007 and 2017, with 82% of census tracts with at least one homicide. The average number of cleared homicides per census tract was 3.9 (SD 3.51) (Fig. 1). The majority of victims were males (81%) and of minority race/ethnicity (75%), with a mean age of 32.4 years old (Table 1). Homicide case clearance was more likely for homicides with female (60.6%) than male (53.9%) victims. Fewer homicides with minority victims were cleared (51.3%) than with white victims (66.7%). Neighborhoods with fewer cleared homicides had higher levels of shots fired per 100,000 population, higher violent crime rates per 100,000 population, higher income inequality, and a higher proportion of residents who identified as minority.

**Table 1:** Individual and neighborhood characteristics by homicide case clearance status

Characteristic	Uncleared	Cleared	Total	*X*^2^
Individual (***N*** = 1320)	n(%)	n(%)	n	***p*** value
Age, mean (SD)	32 (12.4)	32.5 (15.7)		*p* < 0.002
Under 14	6 (1.22)	41 (4.94)	47 (3.5)	
14–17	15 (3.06)	33 (3.98)	48 (3.6)	
18–24	131 (26.7)	222 (26.7)	353 (26.7)	
25–29	89 (18.1)	127 (15.3)	216 (16.3)	
30–34	79 (16.1)	99 (11.9)	178 (13.4)	
35 and older	170 (34.7)	308 (37.11)	478 (36.2)	
Sex				*p* < 0.000
Male	431 (88.1)	639 (76.9)	1070 (81.0)	
Female	58 (11.8)	191 (23.1)	249 (18.8)	
Race				*p* < 0.000
African American	416 (85.0)	576 (69.4)	992 (75.1)	
White	74 (15.0)	254 (30.6)	328 (24.8)	
Ethnicity				*p* < 0.354
Hispanic	32 (6.53)	44 (5.30)	76 (5.76)	
Non-Hispanic	458 (93.5)	786 (94.7)	1244 (94.2)	
**Event**				
Year				*p* < 0.001
2007–2008	146 (29.8)	235 (28.3)	381 (28.8)	
2009–2011	94 (19.1)	193 (23.5)	287 (21.7)	
2012–2014	114 (23.2)	244 (29.4)	358 (27.1)	
2015–2017	136 (27.7)	158 (19.0)	294 (22.2)	
**Neighborhood – Interquartile Range**				
Homicide Rate per 100,000	344.2, 679.2, 984.5	307.2, 498.6, 777.0	160. 6, 344.2, 665.4	
Resident Complaint Rate per 100,000	51.1, 93.7, 162.1	51.3, 93.7, 144.8	52.2, 89.4, 144.5	
Disorder Rate per 100,000	40,855, 53,093, 75,347	35,392, 50,639, 67,889	28,207, 45,357, 65,635	
Violent Crime Rate per 100,000	24.9, 31.2, 46.0	21.4, 27.5, 40.9	13.5, 25.9, 38.4	
Shots Fired per 100,000	4117, 7539, 9509	3079, 4826, 9162	2425, 4252, 8547	
Arrest Rate per 100,000	2053, 6566, 10,122	2037, 5412, 8610	578, 2185, 6566	
GINI	.43, .47, .51	.42, .46, .49	.41, .45, .49	
Percent Minority	71.8, 85.5, 93.8	55.9, 78.0, 92.6	43.6, 71.1, 86.2	

### Multivariable factors associated with homicide case clearance

Three multivariable models were examined: one including all victims from all neighborhoods (Model A); one including homicides of African-American victims only, from all neighborhoods (Model B); and one including all homicides within the upper quartile of neighborhoods with the highest proportion of minority residents (Model C) (Table 2).

**Table 2:** Individual-and community-level variables and homicide case clearance, by Victim/Neighborhood racial/ethnic characteristics, Indianapolis, IN, 2007–2017

	Model A		Model B		Model C	
	All Victims, All Neighborhoods *N* = 1320		African American Victims, All Neighborhoods *n* = 908		Minority Neighborhoods, All Victims *n* = 674	
	OR	Std. Error	OR	Std. Error	OR	Std. Error
**Victim Characteristics**						
Minority race/ethnicity	**.566**	.095	–	–	.896	.264
Male sex	**.576**	.099	**.538**	.119	**.483**	.121
Age, years						
0–13	**3.10**	1.44	**4.29**	2.73	**5.80**	4.46
14–17	1.41	.481	1.54	.577	1.27	.549
25–29	.800	.149	.767	.160	.743	.183
30–34	**.667**	.131	.652	.143	**.508**	.135
35 and older	.862	.135	**.669**	.122	**.654**	.134
**Homicide Year**						
2009–2011	1.32	.227	1.19	.249	1.29	.310
2012–2014	**1.45**	.236	1.12	.214	1.25	.273
2015–2017	.812	.134	**.641**	.125	.820	.185
**Community Characteristics**						
Resident Complaints against Police Rate	1.08	.158	1.33	.225	1.47	.296
Resident Complaints about Community Disorder Rate	.**687**	.122	**.656**	.135	**.611**	.152
Violent Crime Rate	.910	.170	.733	.161	.730	.182
Shots Fired Run Rate	.752	.136	.773	.158	.816	.187
Arrest Rate	**1.65**	.294	**1.93**	.417	**1.75**	.441
Gini Index	.244	.247	.402	.465	.427	.590
Percent Minority Neighborhood	**.511**	.071	**.547**	.087	–	–
Constant	**6.18**	1.55	**4.38**	1.27	**2.94**	1.15

Bold values statistically significant at *p* < 0.05 Reference categories – Age: 18–24 years old; Year: 2007–2008

For Model A, homicides with victims under 14 years of age (OR 3.10; 95% CI, 1.24–7.72; *p* < 0.01) compared to victims 18–24 years old and neighborhoods with higher arrest rates (OR 1.65; 95% CI, 1.16–2.34; *p* < 0.01) compared to those with lower arrest rates were associated with greater odds of homicide case clearance. Homicides with minority (OR 0.566; 95% CI, 0.407–0.787; *p* < 0.01) or male (OR 0.576; 95% CI, 0.411–0.807; *p* < 0.01) victims had a lower odds of case clearance, compared to white and female victims, respectively. Neighborhoods with higher rates of community disorder were associated with a decreased odds of case clearance (OR 0.687; 95% CI, 0.485–0.973; *p* < 0.01), compared to neighborhoods with lower rates of community disorder. Homicides occurring in neighborhoods with a higher proportion of minority residents (OR 0.511; 95% CI, 0.389–0.673; *p* < 0.01) were associated with decreased odds of case clearance.

For Model B, homicide case clearance were almost five times greater for victims less than 14 years of age (OR 4.29: 95% CI, 1.23–14.9; *p* < 0.01) whereas victims ages 35 years and older had a decreased odds of case clearance (OR 0.669; 95% CI, 0.467–0.958; *p* < 0.05), compared to 18–24-year olds. Homicides were less likely to be cleared in 2014–2017 (OR 0.640; 95% CI, 0.437–0.938; *p* < 0.05), compared to 2007 for African American homicide victims. Additionally, neighborhoods with higher levels of resident complaints of community disorder and higher proportions of minority residents were associated with decreased odds of homicide clearance for African American homicide victims (OR 0.656; 95% CI, 0.437–0.985; *p* < 0.01; OR 0.547; 95% CI, 0.400–0.749; *p* < 0.01, respectively).

Lastly, for Model C, victims’ race/ethnicity or year of the homicide were not significantly associated with homicide clearance. Similar to prior models, higher rates of community disorder were associated with a lower odds of homicide case clearance (OR 0.611; 95% CI, 0.375–0.998; *p* < 0.05), compared to neighborhoods with lower resident complaints of community disorder. Similarly, neighborhoods with higher arrest rates were associated with higher odds of homicide case clearance, compared to neighborhoods with lower arrest rates (OR 1.75; 95% CI, 1.07–2.87; *p* < 0.01). Interestingly, the rate of resident complaints against the police only just failed to meet statistical significance and indicates higher rates of resident complaints against the police is associated with higher odds of homicide case clearance (OR 1.47; 95% CI, 0.997–2.19; *p* = 0.51), compared to neighborhoods with a lower rate of resident complaints against the police, respectively.

Neighborhood levels of neighborhood violent crime rates, neighborhood income inequality, and neighborhood shots fired rates did not reach the level of statistical significance across any of the analyses.

## Discussion

The homicide clearance rate across all Indianapolis metropolitan area census tracts was about 40% in 2017, compared to a national homicide clearance rate of about 60% (Investigation FBo, 2016). Homicide clearance rates decreased from 60% since 2007. Moreover, homicide clearances were not equally distributed across all neighborhoods: two elements of community engagement with the police– resident complaints about community disorder and neighborhood arrest rates – were associated with homicide clearance rates, albeit with opposing influences. Higher levels of resident complaints about community disorder were associated with a reduced likelihood of homicide clearance, while higher neighborhood arrest rates were associated with a greater likelihood of homicide clearance. Moreover, an additional police-specific measure of community engagement – resident complaints against police – overall was not associated with homicide clearance, however, was nearly associated with a greater likelihood of homicide case clearance in neighborhoods with higher proportions of residents of color, compared to models that accounted for all victims or only African American victims.

Other individual and neighborhood-level characteristics were associated with homicide clearance, at least in some analyses. Homicide clearance was more likely when the victim was a minor, as child victims are more likely to be killed by a known individual (Regoeczi, 2018). In regards to victim race/ethnicity and neighborhood racial/ethnic composition, homicides were less likely to be solved in communities with higher proportions of minorities, for both minority and white victims. These findings contribute to the prior mixed findings on victim race/ethnicity and neighborhood racial/ethnic composition (Litwin, 2004; Puckett & Lundman, 2003; Lundman & Myers, 2012; Petersen, 2017) and highlights the importance of neighborhood social processes in understanding homicide clearance rates (Regoeczi & Jarvis, 2013; Petersen, 2017).

Among African-American victims, homicide clearance was less likely during the years 2015–2017, compared to 10 years ago. It is plausible these overall declines could be attributed to the national attention on negative police-resident encounters, as prior work indicates publicized cases of police violence decreases residents willingness to call the police, especially in neighborhoods with higher proportions of African American residents (White et al., 2018; Desmond et al., 2016). Our results, however, indicate resident are still calling the police for specific domains of community disorder. Explanations of this finding may lie in the source of residents’ motivations for use of local governmental resources to address community issues. Resident complaints about community disorder may be experienced as direct threats to security and wellbeing – and residents may engage police strategically when it is beneficial to their own or their family’s wellbeing (Bell, 2016; Rios, 2011). This could indicate that even if the overall community is cynical and distrustful about the police and other institutional systems, police are viewed as effective in specific domains (e.g., disturbances and drug dealing), and legal cynicism (a cultural frame in which people perceive the law as illegitimate, unresponsive, and ill equipped to ensure public safety") (Kirk & Papachristos, 2011) is not a simple dichotomy that is either “absent” or “present” (Clampet-Lundquist et al., n.d.). Prior qualitative work demonstrates a person’s willingness to engage the police differs based on sex, age, prior arrest history, and crime type (Bell, 2016; Clampet-Lundquist et al., n.d.). Fear of retaliation can damper cooperation during police investigations (Clampet-Lundquist et al., n.d.), as residents do not trust the police to protect them from violent street crime (Bell, 2016). Residents within disadvantaged communities, however, believe the police make communities safer and should be involved to help resolve neighborhood issues (Bell, 2016; Carr et al., 2007).

Overall, our findings indicate that community-level social processes associated with areas of unsolved homicides are complex, however, our findings support prior research which suggests homicide clearances are influenced by neighborhood dynamics (Regoeczi & Jarvis, 2013; Petersen, 2017). Initiatives to strengthen the police and community ties may be key steps to improve the likelihood of homicide clearance. Evidence suggests community policing is an avenue for police to build trust with residents, improve police legitimacy, and uses problem-solving approaches to reduce crime, fear of crime, and retaliation (Skogan, 1992; Skogan, 2006b; Skogan & Hartnett, 1999; Jones-Webb et al., 2018; Brookman & Innes, 2013). Community policing has also shown success in improving police investigations and cities with the highest homicide clearance rates incorporated strong community policing practices (Skogan et al., 1999; Carter & Carter, 2016). This notion of improved relationships and communication with residents, is an important avenue for homicide detectives as well during the homicide investigation. Family members of homicide victims (i.e., homicide survivors) reported a positive relationship with and trust in police when they felt listened to and had open communication with the homicide detective on their case (Wellman & Meitl, 2020). Unsolved cases leaves survivors to feel that their loved one has been forgotten or overlooked and leaves many feeling dissatisfied with the justice system (Stretesky et al., 2010). Community-wide forums where homicide detectives, homicide survivors, and other important parties come together to discuss the cases, may help family members feel heard, respected, and their family member remembered; which may be as important as solving the case (Wellman & Meitl, 2020; Stretesky et al., 2016).

Beyond the importance of the relationship between the police and residents may be the historical context of the neighborhoods. In Philadelphia, higher levels of violence in 2013 and 2014 occurred predominantly in the same areas that were deemed unworthy of economic investment in the 1937 Home Owners Loan Corporation due to racial and ethnic composition of the residents nearly a century prior (Jacoby et al., 2018). Higher levels of neighborhood violence has been associated with neighborhood fear, dissatisfaction, out-migration, and increased racially segregated poverty (Skogan, 1992; Sampson, 2012). Neighborhoods with higher rates of violence and disorder, often experience higher rates of police presence due to hot spot and broken windows policing (Braga et al., 2012; Braga & Bond, 2008), increasing the likelihood of police interactions and in, some cases, views of decreased police legitimacy (Sherman & Eck, 2002; Kamalu & Onyeozili, 2018). These constellations of findings and associated patterns within communities of high violence and police presence is complex, especially given the historical nature of structural and racial inequalities that these communities have experienced for centuries.

Future work should gather narratives from the neighborhood residents, such as the work on homicide survivors, which highlights the continued trauma and lack of legitimacy in law enforcement when homicide cases go unsolved (Wellman & Meitl, 2020; Stretesky et al., 2016; Stretesky et al., 2010). Additional work should further explore the social dynamics associated with concentrated unsolved homicides to better understand the broader health implications on disadvantaged communities with high levels of violence. Indianapolis has already seen record-breaking levels of homicides in recent years (IndyStar, 2018; IndyStar, 2019), as has been observed in several other metropolitan areas (Rosenfeld et al., 2017). We as a public health community run the risk of continuing the cycle of violence and contributing to health inequalities if we are unable to identify opportunities to improve homicide clearance rates.

## Limitations

Our study took an important first step in exploring the neighborhood social processes associated with unsolved homicides, however, these findings cannot be interpreted as casual mechanisms and is largely exploratory work. Because of this, coefficients in the model and Table 2 should not be interpreted as stand-alone effect estimates, but associations given adjustment for all other model covariates. Additional and more thorough data collection on broader community measures is needed before casual inferences can be made, as our study is likely missing important confounders. For example, although our data demonstrate temporal trends, we do not have longitudinal data that would capture community efforts to improve case clearance rates or identify other socioeconomic or demographic changes that affect resources committed to case clearance. The outcome measure of homicide clearance was gathered from the *Washington Post* and does not include arrests occurring for homicides after 2017 and thus more recent homicides may be falsely categorized as not cleared; however, the first 48–72 h period following a homicide is the most critical to solving the case (Carter & Carter, 2016), and 95% of cases from the National Incident-Based Reporting system were cleared within the first 6 months (Regoeczi et al., 2008). Given the Washington Post does not provide exact data definitions on race/ethnicity and the separate Hispanic category and the assumption race-ethnicity categories are mutually exclusive, we assume White means White-Hispanic. Due to the unknown original source of race/ethnicity coding we cannot draw conclusions for more nuanced ethnicity and racial groups. In an attempt to account for potential spatial autocorrelation, we ran sensitivity analysis by including the *X* and *Y* coordinates for each homicide instead of nesting the incidents within census tracts, and results from those models were subsequently unchanged from the results presented in this article (Wheeler, 2018). Additionally, these analyses do not control for incident circumstances such as perpetrator, motive, weapon type, seasonality, or investigative details; and could be important confounding variables. This study is the first to empirically test resident complaints against the police and homicide clearance, though not all residents with a negative interaction make a formal report. Thus, we could be missing individuals with the highest levels of cynicism. Likewise, not all reports of community disorder may be reported but rather both reflect these factors combined with the community members’ willingness to report. Given the complexity of what these measures capture, we frame our findings based on how these might indicate possible mechanisms and to generate hypotheses for future research.

## Conclusion

This study identified key neighborhood social processes associated with unsolved homicides within Indianapolis neighborhoods. In particular, resident complaints of community disorder were associated with lower homicide clearance, whereas resident complaints against the police were nearly associated with greater clearance. Programs aimed at building community engagement and improving relationships between residents and the police may improve neighborhood clearance rates, lower violence, and improve the health of these communities.

## Data Availability

The datasets used in this manuscript are available from publicly available data warehouses or available from the corresponding author on reasonable request.
